# HIV/SIV Infection Primes Monocytes and Dendritic Cells for Apoptosis

**DOI:** 10.1371/journal.ppat.1002087

**Published:** 2011-06-23

**Authors:** Mireille Laforge, Laure Campillo-Gimenez, Valérie Monceaux, Marie-Christine Cumont, Bruno Hurtrel, Jacques Corbeil, John Zaunders, Carole Elbim, Jérôme Estaquier

**Affiliations:** 1 INSERM U955, Faculté Créteil Henri Mondor, Créteil, France; 2 Unité de Physiopathologie des Infections Lentivirales, Institut Pasteur, Paris, France; 3 Université Laval, Centre de Recherche en Infectiologie, Québec, Canada; 4 St Vincent's Centre for Applied Medical Research, St Vincent's Hospital, Darlinghurst, Australia; 5 Université Paris Descartes, UMR S 872, Paris, France; 6 Assistance Publique-Hôpitaux de Paris, Hôpital Henri Mondor, Créteil, France; SAIC-Frederick, United States of America

## Abstract

Subversion or exacerbation of antigen-presenting cells (APC) death modulates host/pathogen equilibrium. We demonstrated during *in vitro* differentiation of monocyte-derived macrophages and monocyte-derived dendritic cells (DCs) that HIV sensitizes the cells to undergo apoptosis in response to TRAIL and FasL, respectively. In addition, we found that HIV-1 increased the levels of pro-apoptotic Bax and Bak molecules and decreased the levels of anti-apoptotic Mcl-1 and FLIP proteins. To assess the relevance of these observations in the context of an experimental model of HIV infection, we investigated the death of APC during pathogenic SIV-infection in rhesus macaques (RMs). We demonstrated increased apoptosis, during the acute phase, of both peripheral blood DCs and monocytes (CD14^+^) from SIV^+^RMs, associated with a dysregulation in the balance of pro- and anti-apoptotic molecules. Caspase-inhibitor and death receptors antagonists prevented apoptosis of APCs from SIV^+^RMs. Furthermore, increased levels of FasL in the sera of pathogenic SIV^+^RMs were detected, compared to non-pathogenic SIV infection of African green monkey. We suggest that inappropriate apoptosis of antigen-presenting cells may contribute to dysregulation of cellular immunity early in the process of HIV/SIV infection.

## Author Summary

Antigen-presenting cells (APCs) are critical for both innate and adaptive immunity. They have a profound impact on the hosts' ability to combat microbes. Dysfunction and premature death by apoptosis of APCs may contribute to an abnormal immune response unable to clear pathogens. Circulating blood monocytes exhibit developmental plasticity, with the capability of differentiating into either macrophages or dendritic cells (DCs), and they represent important cellular targets for HIV-1. We report that HIV infection renders monocytes/macrophages and DCs in vitro more prone to undergo apoptosis and this heightened susceptibility is associated with changes in the expression of anti- and pro-apoptotic molecules. Our results show that during the acute phase of SIV-infection of rhesus macaques, monocytes and DCs are more prone to die by apoptosis. They express lower levels of Mcl-1 and FLIP proteins, two anti-apoptotic molecules, but higher expression of the active form of Bax and Bak, the gatekeepers of the mitochondria, major sensor of the apoptotic machinery. Because the early events are important in the pathogenesis of this disease, early death of APCs should play a major role leading to the defective immune response. Strategies aimed at preventing death of APCs could be beneficial in helping the immune response to fight HIV-1.

## Introduction

Monocytes originating from the bone marrow are released into peripheral blood, where they circulate for several days before entering tissues, and replenish tissue macrophage populations in the steady state. Monocytes constitute a considerable systemic reservoir of myeloid precursors. Monocytes exhibit developmental plasticity, with the capability of differentiating into either macrophages or dendritic cells (DCs) *in vitro* depending on the cytokine milieu. They can enter in lymphoid tissues during inflammation and give rise to macrophages and inflammatory DCs [1], [2], [3]. Classical DCs represent a distinct lineage of myeloid cells that are also present in the blood and can migrate into the tissues [3]. Mononuclear phagocytes are critical for both innate and adaptive immunity. Recruited to inflammatory sites, cDCs, inflammatory DCs and macrophages play a critical role in the protection against pathogens [3], [4], [5], [6].

Mononuclear phagocytes and DCs which express CD4 receptor and chemokine co-receptors represent important cellular targets for human immunodeficiency virus type-1 (HIV-1). Circulating monocytes can be latently infected and productive infection can be initiated during differentiation into macrophages [7], [8]. Mononuclear phagocytes are rendered defective specifically by the envelope glycoprotein that impairs maturation and cytokine secretion [9], [10]. This contributes to the development of immune deficiency observed during HIV infection [11], [12], [13], [14].

The most striking feature of AIDS is the increased death and progressive depletion of CD4^+^ T lymphocytes which leads to immunodeficiency [15]. CD4^+^ T cells from HIV-infected individuals and SIV-infected rhesus macaques are more sensitive to undergo apoptosis due to the effects of death-receptors [16], [17], [18], [19], [20], [21], [22], [23], [24], [25]. Moreover, in the absence of viral replication, HIV or SIV primes CD4+ T cells for apoptosis *in vitro* [25], [26], [27]. In contrast, the impact of HIV on apoptosis of monocytes and DCs has not been extensively studied.

Monocytes, but not macrophages, are prone to undergo apoptosis after death-receptor ligation [16], [28], [29], [30], [31]. Death receptors include Fas/CD95, TRAIL-Receptor, and TNF-Receptor. The engagement of death-receptors by their counterparts, FasL, TRAIL and TNF, either in soluble form or at the membrane surface of the cells, induce death-signaling cascades. The molecular ba.sis of resistance to death-receptors-mediated apoptosis involves FLIP (cellular-FLICE-inhibitory protein expressed during differentiation of APCs [31], [32], [33]), an inhibitor of the DISC (death-inducing signaling complex) [34]. Moreover, apoptosis initiated by growth factor deprivation can be prevented by a decoy-receptor that blocked Fas and FasL interaction [31], [35], [36], and mice carrying functional mutations of Fas-FasL displayed elevated monocytic cell counts [37]. In addition, to the extrinsic pathway that involves death-receptors and their counterparts, apoptosis regulation in mononuclear phagocytes includes also the intrinsic pathway. Thus, among the anti-apoptotic members, Mcl-1 predominates in differentiated cells [38]. Mitochondrial outer membrane integrity is highly controlled, primarily through interactions between pro- and anti-apoptotic of the members of the Bcl-2 protein family. On activation, Bax and Bak proteins undergo extensive conformational changes leading to mitochondria permeabilization and cell death [39].

Subversion of monocyte apoptosis by intracellular bacteria or parasites is used by pathogens to favor their own replication and dissemination within the host when death is inhibited [40], [41], [42], [43], [44], [45], [46]. In contrast, massive cell death of infected macrophages induced by the Ebola virus contributes to pathogenesis by abolishing innate immunity [47]. Several viral infections are also associated with the death of DCs [45], [48], although DCs, unlike monocytes, are mostly resistant to FasL-induced cell death [33], [49], [50], [51], [52].

Differentiated macrophages infected by HIV *in vitro* are more resistant to TRAIL-mediated cell death triggered by the envelope protein [53] whereas another report suggests that HIV-infected macrophages are more prone to undergo apoptosis [54]. In the peripheral blood of chronically HIV-infected individuals and SIV-infected rhesus macaques (RMs), reduced numbers of DCs are found [55], [56], [57], [58], [59], [60], [61] consistent with increased death of those cells [62], [63], [64]. Furthermore, in chronically SIV-infected RMs, massive turnover of peripheral monocytes undergoing apoptosis have been reported [65]. In viremic HIV-infected individuals it has been shown that both spontaneous and IFN-ã-induced monocyte cell death are elevated compared to controls [66] although another report describes monocytes resistant to cell death, associated with antiapoptotic gene profiles [67]. However, little information exists on the precise molecular mechanisms involved and only few studies have assessed these processes early after infection.

Indeed, an increasing amount of evidence suggests that the acute phase dictates the rate of progression towards AIDS. Experimental infection of RMs of Chinese origin is an extremely valuable model to investigate these early events [22], [68], [69], [70], [71]. The aims of the present study were to determine whether HIV/SIV infection early after viral exposure sensitizes mononuclear phagocytes for apoptosis and to elucidate the molecular mechanisms behind the process. We assessed the relevance of apoptosis inducing processes during the acute phase of pathogenic lentiviral infection of RMs.

We demonstrated that *in vitro* and *in vivo,* monocytes and DCs exposed to HIV/SIV are sensitized to death-receptors ligation-mediated cell death. Among death-ligands, TRAIL and FasL were the most potent at promoting apoptosis of monocytes and DCs, respectively. Lower amounts of FLIP and Mcl-1 and an increase in the levels of the active form of Bax and Bak proteins were found. A broad caspase inhibitor prevented cell death and increased the number of TNF-α productive mononuclear cells. Thus, the inappropriate death of circulating mononuclear phagocytes during the acute phase could favor the development of a state of immunodeficiency.

## Results

### HIV-1 infection impairs cytokine production and maturation of MØ and DCs

**Figure 1:**
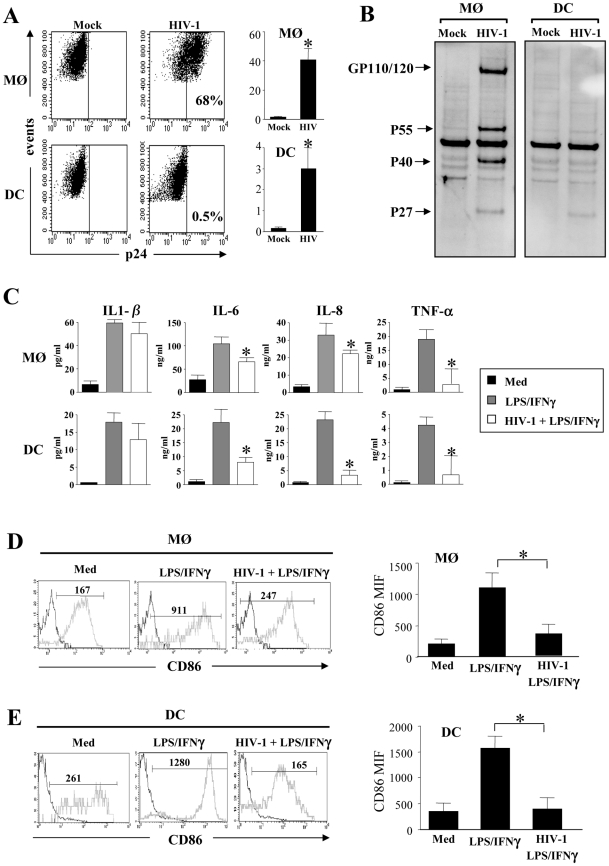
Impact of HIV infection on cytokine secretion and maturation of monocyte-derived macrophages and monocyte-derived DCs. (**A** and **B**) HIV-1 infection of monocyte-derived macrophages (MØ) or monocyte-derived DCs. Cells were infected at day 1 after the initiation of APC differentiation without (Mock) or with the R5 HIV-1_Bal_ (100 pg/ml of p24). At day 5, (**A**) the percentage of p24^+^ cells was determined by flow cytometry. Values shown are means ± SEM (n = 6). Significant differences are indicated by an asterisk (p<0.05). (**B**) HIV viral proteins were detected by western blotting using HIV-1^+^ sera. (**C**) HIV-1 decreases pro-inflammatory cytokines production. Cells at day 5 were stimulated with LPS (10 ng/ml) and IFN-γ (10^3^ U/ml) overnight. Cells incubated in the absence of R5 HIV-1_Bal_ and in the absence of stimulation represent the negative control (Med). Supernatants were collected and assessed for the presence of IL-1β, IL-6, IL-8, and TNF-α by flow cytometry using bead array. Values shown are means ± SEM (n = 3). Significant differences are indicated by an asterisk (p<0.05). (**D** and **E**) HIV-1 decreases CD86 expression at the surface of stimulated (**D**) MØ or (**E**) DCs. Cells were stained with specific CD86 mAbs, and cell surface density was assessed by flow cytometry. One representative experiment out of three is shown; the mean of fluorescence intensity is indicated. CD86 expression values shown are means ± SEM (n = 3). Significant differences are indicated by an asterisk (p<0.05).

Blood monocytes are non-cycling, non-proliferating cells incapable of supporting viral replication. Indeed, establishment of productive infection coincides with entry into G1/S phase of the cell cycle [72], and GM-CSF is one of the main cytokines that promotes and sustains productive infection [7], [8], [73], [74], [75]. We infected monocyte-derived macrophages (MØ)- and monocyte-derived DCs (immature DCs) during differentiation. One day after the process of differentiation was initiated, with either GM-CSF and IL-6 for MØ or GM-CSF and IL-4 for DCs, the R5 HIV-1 tropic strain, HIV-1BaL was added to simulate the presence of HIV-1 during the maturation process. This contrasts with the addition of virus at the end of the differentiation process utilized in most, if not all, published studies [53], [76], [77]. After 5 days, we assessed the percentage of infected MØ and DCs based on intracellular p24 staining by flow cytometry. As expected, we found that the percentage of MØ infected by the R5 tropic strain HIV-1_BaL_ was higher than DCs (**Figure 1A**). The percentage of HIV-infected MØ varied (40%±7) among individual preparations, whereas DCs from the same individuals displayed less than 3%±1 of infected cells, consistent with previous reports [13], [78]. To confirm intracellular staining of p24 antigen, the cells were lysed and western blots performed to detect the profile of viral antigens, using sera from HIV-infected individuals. In MØ, we observed a typical profile displaying both envelope glycoprotein and gag protein, whereas none of these bands were clearly observed in DCs (**Figure 1B**). We then assessed the capacity of MØ and DCs to produce cytokines and express co-stimulatory molecules in response to LPS and IFN-γ. We found that activated-MØ as well as activated-DCs, incubated in the presence of HIV-1, secreted less pro-inflammatory cytokines such as IL-6, IL-8 and TNF-α as compared to uninfected cells. No difference was observed for IL-1β secretion (**Figure 1C**). Moreover, stimulation with LPS and IFN-γ induced lower expression of the co-stimulatory molecule CD86 (**Figure 1D, E**) and the maturation marker CD83 (data not shown), at the surface of HIV-infected cells as compared to uninfected cells (CD86 mean expression, MØ: 350±150 vs 1090±230; DCs: 400±220 vs 1570±230). Thus, HIV infection during the process of APC differentiation impacted cytokine secretion and cellular maturation.

### HIV-1 infection sensitizes MØ and DCs to death receptors-mediated apoptosis by downregulating the expression of FLIP

**Figure 2:**
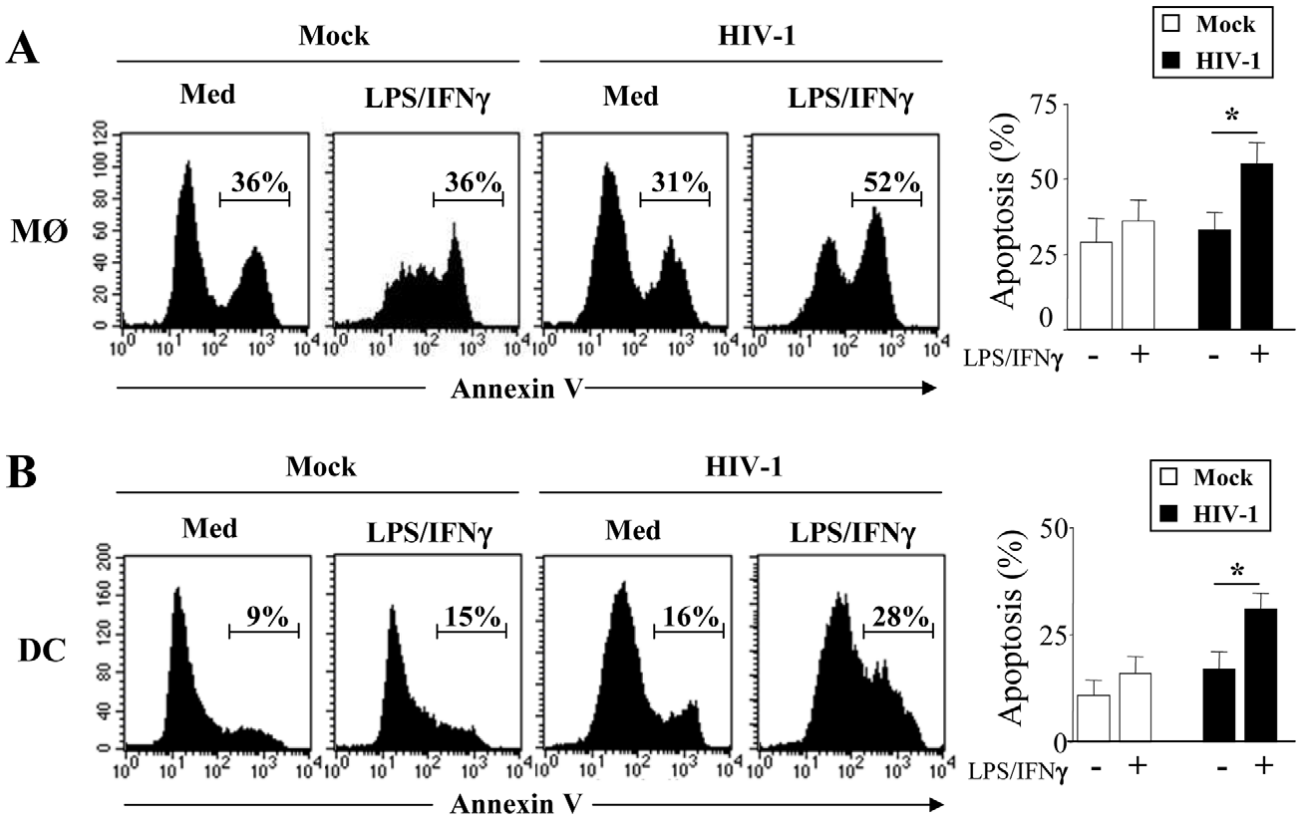
HIV-1 infected monocyte-derived macrophages and monocyte-derived DCs undergo apoptosis after stimulation. (**A**) Monocyte-derived macrophages (MØ) or (**B**) monocyte-derived DCs were incubated with R5 HIV-1_Bal_ (100 pg/ml of p24) or Mock control at day 1 after the initiation of APC differentiation and then stimulated at day 5 without or with LPS (10 ng/ml) and IFN-γ (10^3^ U/ml) overnight. Apoptosis was assessed by flow cytometry using FITC-Annexin-V. One representative experiment out of three is shown. Percentages of apoptotic cells shown are means ± SEM (n = 3). Statistical significant differences, as compared to non stimulated cells, are indicated by an asterisk (p<0.05).

**Figure 3:**
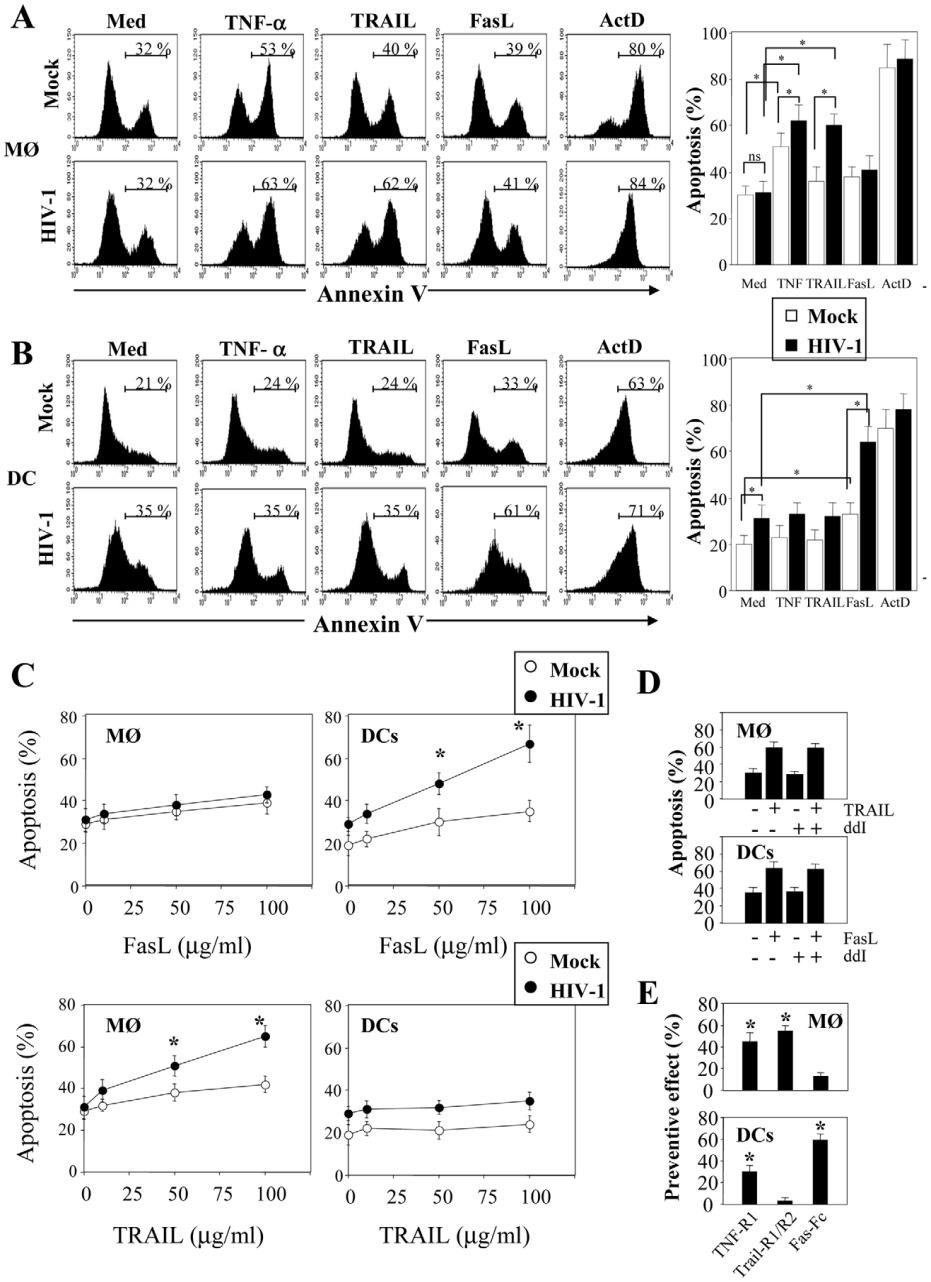
HIV-1 sensitizes monocyte-derived macrophages and monocyte-derived DCs for Death receptor ligands. (**A**) Monocyte-derived macrophages (MØ) or (**B**) monocyte-derived DCs were incubated with R5 HIV-1_Bal_ (100 pg/ml of p24) or Mock at day 1 after the initiation of APC differentiation. At day 5, the cells were then cultured overnight in the absence or presence of recombinant TNF-α, TRAIL and FasL (100 ng/ml). A positive control of cell death was performed in the presence of Actinomycin D (10 µg/ml). Apoptosis was determined by flow cytometry using FITC-labeled Annexin V. Percentages of apoptotic cells shown are means ± SEM (n = 3). Statistical significant differences are indicated by an asterisk (p<0.05). (**C**) Dose response of FasL and Trail. Percentages of apoptotic cells shown are means ± SEM (n = 3). Statistical significant differences as compared to untreated cells are indicated by an asterisk (p<0.05). (**D**) Cells, before infection, were incubated in the absence or presence of ddI (5 µM). At day 5, the cells were then cultured overnight in the absence or presence of either TRAIL or FasL (100 ng/ml). (**E**) Cells incubated with R5 HIV-1_Bal_ were stimulated at day 5 with LPS (10 ng/ml) and IFN-γ (10^3^ U/ml) overnight in the absence or presence of death receptor antagonists: TNF-R1, TRAIL-R1/TRAIL-R2, Fas-Fc (10 µg/ml). Preventive effect was calculated as follows: ((% of MØ/DC apoptosis - % of MØ/DC apoptosis in the presence of decoy receptors)/(% of MØ/DC apoptosis)) X 100. Values are means ± SEM (n = 3). Statistical significant differences as compared to untreated cells are indicated by an asterisk (p<0.05).

We then examined whether MØ and DCs were more prone to die at day 5 post-infection with HIV-1_BaL_. After stimulation with LPS and IFNγ, we observed a significant increase in the percentage of apoptotic cells from HIV-infected culture as compared to non-infected cells (MØ, 55%±7 vs 31%±3.8, p<0.01; DCs, 36%±7 vs 16%±3.9, p<0.01). No major difference was observed after stimulation of uninfected cells (**Figure 2A, B**). The extrinsic apoptotic pathway involves members of the death-receptor family including CD95 (Fas), TNF-R and DR4/DR5 (Trail-R1/R2) [79]. Upon ligation of these death-receptors by their ligands, the association of the adaptor molecule FADD with the initiator caspases forms a death-inducing signaling complex (DISC) leading to apoptosis [80]. We assessed whether MØ and DCs in the presence of HIV-1BaL are sensitive to death-receptor ligands including TNF-α, TRAIL and FasL. Actinomycin D (Act D) was used as a positive control for cell death. First, both uninfected and HIV-infected MØ (51±6% and 62±7%, respectively) were more sensitive to undergo apoptosis in response to TNF-α in comparison with the medium alone (30±4% and 31±5%, respectively) whereas no similar effect was observed on DCs (**Figure 3A, B**). Second, MØ infected with HIV-1_BaL_ were more prone to die in response to TRAIL as compared to non-infected cells (60±5% versus 36±6%) (**Figure 3A**) but no difference was observed for FasL (38±4% versus 41±6%). Finally, HIV-1 infection increased the sensitivity of DCs to die spontaneously (20±4% uninfected versus 31±6% in infected DCs) and after FasL-ligation (64±7% versus 31±6% in medium alone) but not to the binding of TRAIL (32±6%) (**Figure 3B**). Apoptosis was dependent on the amount of death ligands (**Figure 3C**).

In order to determine if viral replication was necessary for sensitization to apoptosis, we treated the cells with ddI (5 µM, a dose that blocks viral replication in MØ; <5% of p24^+^). Our studies showed that in the presence or absence of ddI, both MØ and DCs remain sensitive to TRAIL and FasL, respectively (**Figure 3D**). Furthermore, we assessed whether stimulation with LPS/IFN-γ-mediated apoptosis may be modulated by antagonists to death ligands using decoy receptors. We demonstrated that decoy receptors of TNF (TNF-R1), and TRAIL (TRAIL-R2/TRAIL-R1), reduced monocyte cell death-mediated by LPS/IFN-γ stimulation, whereas decoys receptors of Fas (Fas-Fc) and TNF (TNF-R1) reduced DCs cell death (**Figure 3E**). Thus, despite the fact that soluble TNF-α has no effect on DCs, TNF-R1 partially inhibits cell death. Altogether, these results indicated that HIV induces APC apoptosis after death-receptors ligation.

**Figure 4:**
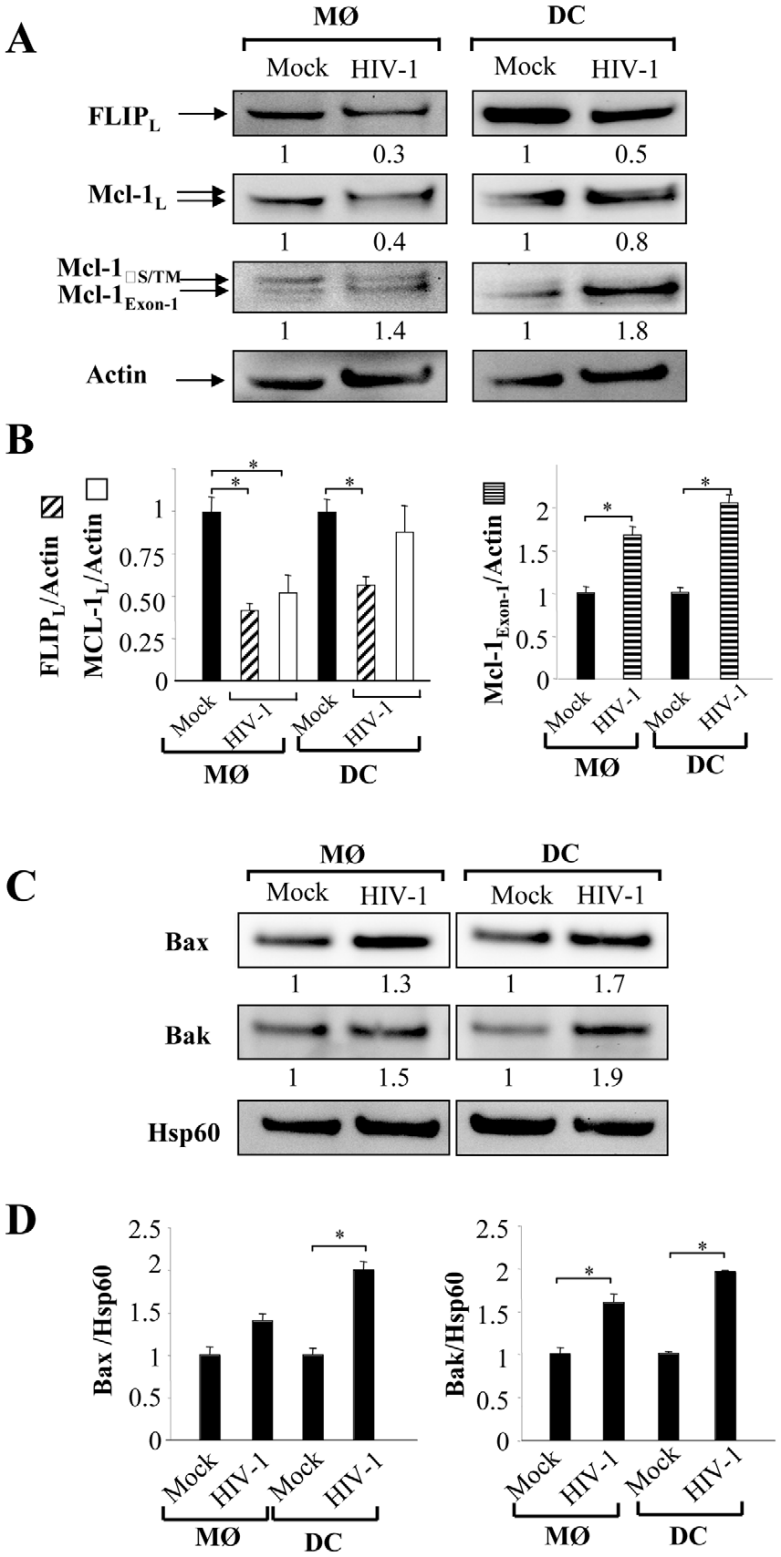
Expression of pro- and anti-apoptotic molecules in HIV-1 infected monocyte-derived macrophages and monocyte-derived DCs. (**A**) Monocytes-derived MØ or monocytes-derived DCs were incubated with R5 HIV-1_Bal_ (100 pg/ml of p24) or Mock at day 1 after the initiation of APC differentiation. At day 5, the cells were lysed and then the proteins were detected by immunoblotting with specific antibodies against FLIP and Mcl-1. Actin was used as a control for equal protein loading. Values represent the ratio of the protein bands normalized with respect to the loading control, analyzed with GeneTools (SynGene). Mock (non-infected cells) was considered arbitrary equivalent to 1, and bands are compared between (Mock) and HIV-infected cells (HIV). (**B**) Ratio of FLIP, Mcl-1 and Mcl-1_Exon-1_ proteins. Bars show the mean ± SEM of three independent experiments. Statistical significant differences as compared to non-infected cells are indicated by an asterisk (p<0.05). (**C**) Expression of pro-apoptotic Bax and Bak molecules in the enriched mitochondrial fraction. Hsp60 was used as a control for equal protein loading. Values represent the ratio of the protein bands normalized with respect to the loading control (Hsp60), analyzed with GeneTools (SynGene). Mock (non-infected cells) was considered arbitrary equivalent to 1, and bands are compared between (Mock) and HIV-infected cells (HIV). (**D**) Ratio of Bax and Bak proteins. Bars show the mean ± SEM of three independent experiments. Statistical significant differences as compared to non-infected cells are indicated by an asterisk (p<0.05).

Since cells were more sensitive to undergo apoptosis, we next assessed whether this effect was related either to a modulation in the expression of death-receptors or in the regulation of the signaling pathway. Although, MØ and DCs exhibited a greater sensitivity to die in the presence of death ligands, we did not observe any modulation of death-receptor expression, including TRAIL-R1 and –R2 and Fas/CD95 on cells infected with HIV-1_Ba-L_ compared to uninfected cells (data not shown). The molecular basis of resistance to death-receptors-mediated apoptosis involves the expression of FLIP (cellular-FLICE-inhibitory protein), which is an inhibitor of the DISC (death-inducing signaling complex) [34], and is expressed during differentiation of APCs [31], [32], [33]. Therefore, we analyzed the expression of FLIP in HIV-infected MØ and DCs. We observed that FLIP expression is detectable by western blot at day 5 in both uninfected MØ and DCs but decreased in HIV-infected cells (**Figure 4A**). Thus, the amount of FLIP decreased by 57%±4 in MØ and 46%±5 in DCs following HIV infection (**Figure 4B**). Altogether, our data suggest that HIV-1 infection increased the propensity of mononuclear phagocytes to undergo apoptosis in response to death-ligands, possibly due to a decrease in the amount of FLIP. This process occurred independently of any modulation of death receptor expression.

### HIV modulates the balance between pro- and anti-apoptotic members of the Bcl-2 family in MØ and DCs

The molecular basis of macrophage resistance to apoptosis includes the expression of the anti-apoptotic Bcl-2 family members, among which Mcl-1 predominates in differentiated cells [38]. In the absence of growth receptor engagement, Mcl-1 is degraded by the ubiquitin-proteasome pathway [81], [82], [83] or cleaved by proteases [84], [85]. We found a 50% decrease in expression of Mcl-1 protein in infected-MØ, which was not observed in DCs (**Figure 4A**). In addition, SDS-PAGE analysis revealed that Mcl-1 migrated as a doublet suggesting the presence of phosphorylated Mcl-1, primed by GSK-3, on threonine 163. This phosphorylated form undergoes accelerated degradation [81], [83]. In HIV-infected MØ, this change in MCL isoforms was clearly observed compared to uninfected cells, whereas no difference was observed for DCs (**Figure 4A**). Additional bands of approximately 34 KDa on western blots probed with Mcl-1 antibody were also detected (**Figure 4A**). These product bands correspond to different translational products (Mcl-1_S/ΔTM_ versus Mcl-1_Exon-1_). It is important to note that Mcl-1_Exon-1_ is pro-apoptotic [38]. The amount of Mcl-1_Exon-1_ protein was clearly enhanced in DCs cultured in the presence of HIV-1_BaL_ (fold increase 2.1) as well as in MØ (fold increase 1.7) (**Figure 4B**).

Members of the Bcl-2 protein family, in particular Bax and Bak proteins play a critical role in controlling apoptosis [39]. To assess the early commitment of Bax and Bak activation, we subfractionated the cells to isolate a mitochondria-enriched fraction. At day 5 of culture, we observed higher amounts of Bax and Bak proteins within the enriched mitochondrial fraction derived from HIV-1_BaL_-infected MØ and DCs compared to uninfected cells (**Figure 4C and D**). Membrane insertion of Bax and Bak supported a dynamic model in which mitochondria is a central sensor. Taken together, these results suggest that HIV shifts the balance towards pro-apoptotic molecules rendering APCs more sensitive to death stimuli.

### Blood monocytes and DCs undergo apoptosis during primary SIV-infection of Rhesus macaques

**Figure 5:**
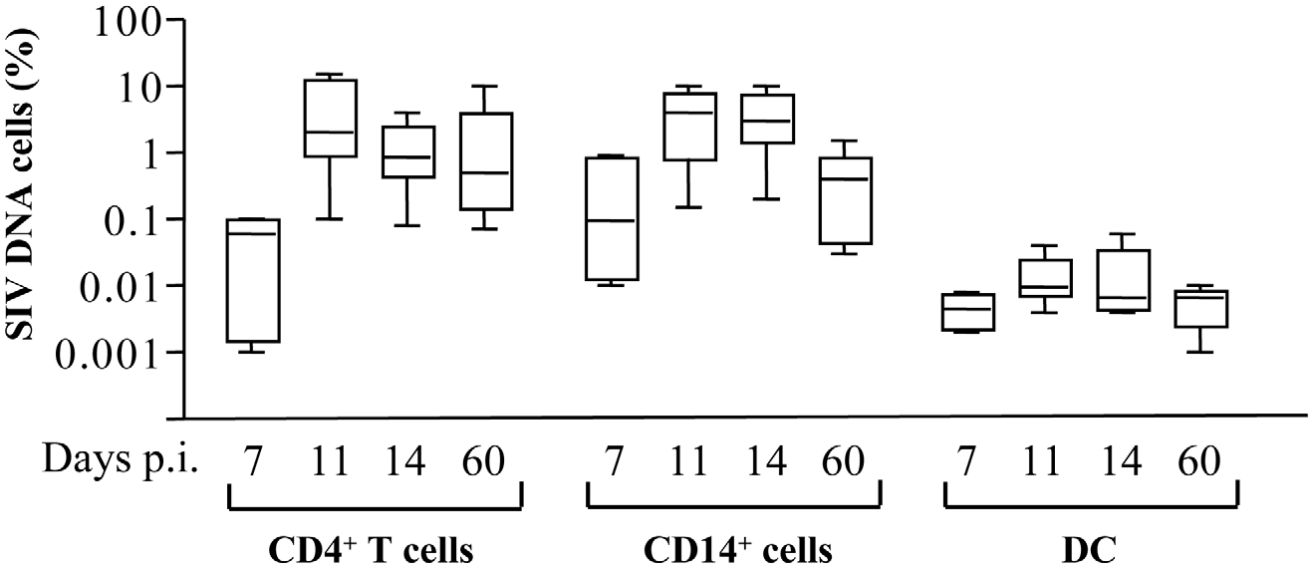
Frequency of SIV-DNA^+^ cells in SIV-infected macaques. Frequency of SIV-DNA^+^ CD4^+^ T cells, CD14^+^ cells and DCs. Prism version 3.0 (GraphPad Software) was used to calculate means ± SD at days 7, 11, 14 and 60 post-infection.

Early events during the acute phase of SIV infection are critical in determining the onset of AIDS, we therefore investigated APC death in RM during acute SIV infection. We analyzed the percentage of monocytes CD14^+^ that were infected compared to CD4^+^ T cells in peripheral blood (the purity was more than 98% for HLA-DR^+^CD14^+^ cells as well for CD4^+^ T cells after cell sorting). HIV-1 has been reported to be isolated from CD14^+^ monocytes of patients under HAART, indicating that monocytes are competent for HIV infection [86]. Moreover, because monocytes circulate in the blood for only a few days before differentiating into macrophages in tissues, they represent important cells in viral dissemination. The frequency of monocytes and CD4^+^ T cells harboring proviral DNA was quantified using a nested SIV PCR assay in limiting dilutions of purified cells [22]. The frequency of SIV-DNA positive monocytes increased and peaked at days 11–14 (day 11, mean: 3.8±1.3; day 14, 3.6±1.2 of monocytes were infected) which is equivalent to the frequency found in CD4^+^ T cells (day 11, mean: 4.8±2.1 and day 14, 1.4±0.4) (**Figure 5**). Thereafter, the frequencies of SIV-infected monocytes decreased (mean: 0.5±0.18). The dynamics of SIV-DNA is consistent with viral load (viral RNA) measured in the plasma (data not shown) [22]. Unlike monocytes and CD4^+^ T cells, the frequency of SIV-DNA in myeloid DCs (HLA-DR^+^CD11^+^) was extremely low during the acute phase of infection (day 11, mean: 0.015±0.004; day 14, 0.015±0.008 of DCs were infected) consistent with a previous report [87].

**Figure 6:**
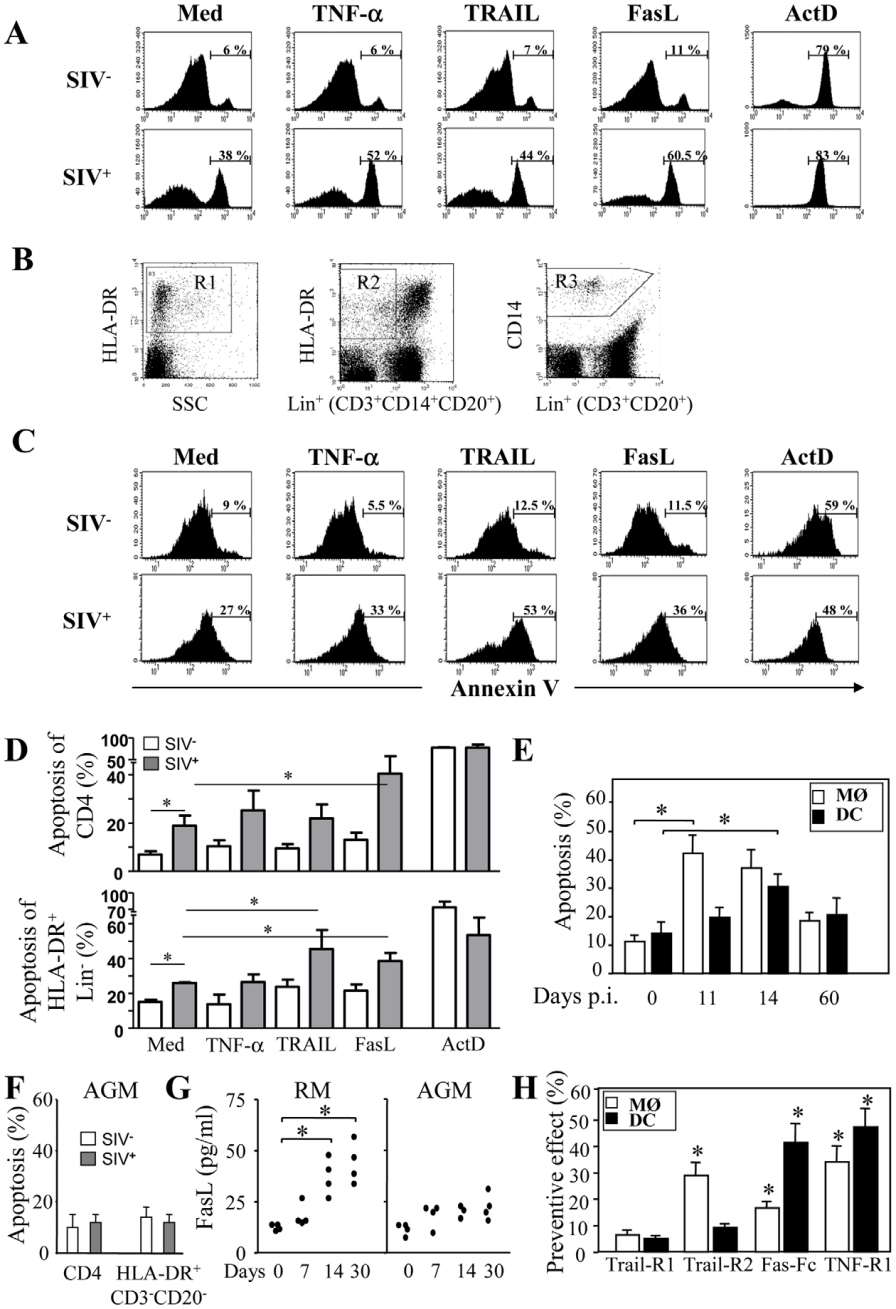
Increased apoptosis of monocytes and DCs during primary SIV infection. PBMC from healthy (**SIV^−^**) and SIV-infected RM at day 14 (**SIV^+^**) were incubated overnight in the absence or presence of death ligands. (**A**) The percentages of apoptotic CD4^+^ T cells were analyzed by flow cytometry using FITC-labeled Annexin V. (**B**) Gating strategy to analyze apoptotic HLA-DR^+^ and CD14^+^ cells. Cells were first analyzed on HLA-DR *versus* SSC (gate R1) and HLA-DR *versus* Lin+(CD3^+^CD14^+^CD20^+^) cells (gate R2), or CD14 *versus* Lin+(CD3^+^CD20^+^) cells (gate R3) (**C**) The percentages of apoptotic HLA-DR^+^Lin^−^ (CD3^−^CD14^−^CD20^−^) cells was determined by flow cytometry using FITC-labeled Annexin V gated on R1 and R2; (**D**) Percentage of apoptotic CD4^+^ and HLA-DR^+^Lin^−^ cells. Values shown are means ± SEM (n = 4 for SIV^−^ and SIV^+^); Significantly different compared to medium controls (*, p<0.05). (**E**) Percentage of apoptotic monocytes (MØ) and DCs at days 0, 11, 14 and 60; values are means ± SEM (n = 6); Significantly different from day 0 (*, p<0.05). (**F**) PBMC from healthy and SIV-infected AGM at day 14. The percentages of apoptotic CD4^+^ T cells and HLA-DR^+^CD3^−^CD20^−^ cells were analyzed by flow cytometry using FITC-labeled Annexin V. (**G**) Quantitation of FasL in the sera of SIV-infected RMs and AGM at different time points post-infection. Statistical significant differences as compared to day 0 are indicated by an asterisk. (**H**) Preventive effect of death receptor antagonists. PBMC from SIV-infected RMs (day 14) were incubated overnight with antagonists of death receptors: TRAIL-R1, TRAIL-R2, Fas-Fc and TNF-R1 (10 µg/ml). Apoptosis of monocytes and DCs was quantified using FITC-Annexin-V. Preventive effect was calculated as follows: ((% of MØ/DC apoptosis - % of MØ/DC apoptosis in the presence of decoy receptors)/(% of MØ/DC apoptosis)) X 100. Values are means ± SEM (n = 5); Significantly different from samples incubated with medium alone (*, p<0.05).

We quantified the percentages of dying HLA-DR^+^CD3^−^CD20^−^ and CD4^+^ T cells before and after incubation with death-ligands by monitoring FITC-labeled annexin V. As previously shown [21], [25], [88], CD4^+^ T cells derived from SIV-infected RMs at the peak of viral replication (day 14) were prone to undergo apoptosis spontaneously and after FasL ligation as compared to Trail or TNF-α ligation (**Figure 6A and D**) and consistent with other studies [89]. Unlike CD4^+^ T cells, among death-ligands, TRAIL and FasL were the most potent ligands to promote apoptosis of HLA-DR^+^CD3^−^CD20^−^ at day 14 (**Figure 6B, C and D**). We then demonstrated that early after infection both monocytes (HLA-DR^+^CD14^+^) and DCs (Lin^−^HLA-DR^+^CD11c^+^CD123^−^) are more prone to undergo apoptosis spontaneously (**Figure 6E**). Thereafter, the levels of apoptosis decreased to reach those observed before infection (**Figure 6E**). In non pathogenic SIV-infected African green monkeys (AGM), apoptosis of CD4 and of HLA-DR^+^CD3^−^CD20^−^ cells at day 14 was similar to the level observed from healthy monkeys (**Figure 6F**) consistent with the absence of apoptosis reported in this non pathogenic primate model, despite a similar level of viral replication comparable to RMs [23], [70], [88]. The biologically active forms of death ligands include both a soluble and a membrane bound form. Therefore, we quantified the presence of death ligands in the sera of SIV-infected monkeys. We found, concomitant with the increase of cell death in RMs, higher levels of FasL two weeks post-infection (**Figure 6G**). In contrast, we did not observe any increase in the levels of FasL in SIV-infected AGM (**Figure 6G**). We have reported during the acute phase the absence of TNF-α detection in the sera of both SIV-infected species [90], [91]. Although, we were unable to detect soluble TRAIL in the sera of SIV-infected monkeys due to the unavailability of appropriate reagents for its detection in non-human primates (data not shown), it has been reported that there is increased expression of Trail mRNA in SIV-infected RMs [92].

To assess the impact of soluble and membrane forms of death ligands, we investigated whether apoptosis of monocytes and DCs from SIV-infected RM may be modulated by antagonists to death ligands using decoy receptors. We demonstrated that decoy receptors of TNF (TNF-R1) and TRAIL (TRAIL-R2 but not TRAIL-R1), and to a lesser extent decoy receptor of Fas (Fas-Fc), reduced monocyte cell death, whereas decoys receptors of Fas and TNF (TNF-R1) reduced DCs cell death (**Figure 6H**). Interestingly, despite the fact that soluble TNF-α has no effect, antagonist antibodies partially inhibited death suggesting that TNF-α at the cell surface may participate in the death of APCs [93]. These results suggest that apoptosis of mononuclear phagocytes involved death-receptors and their counterparts.

### Apoptosis of blood mononuclear cells during primary SIV infection involves a dysregulation in the balance of pro- and anti-apoptotic molecules

**Figure 7:**
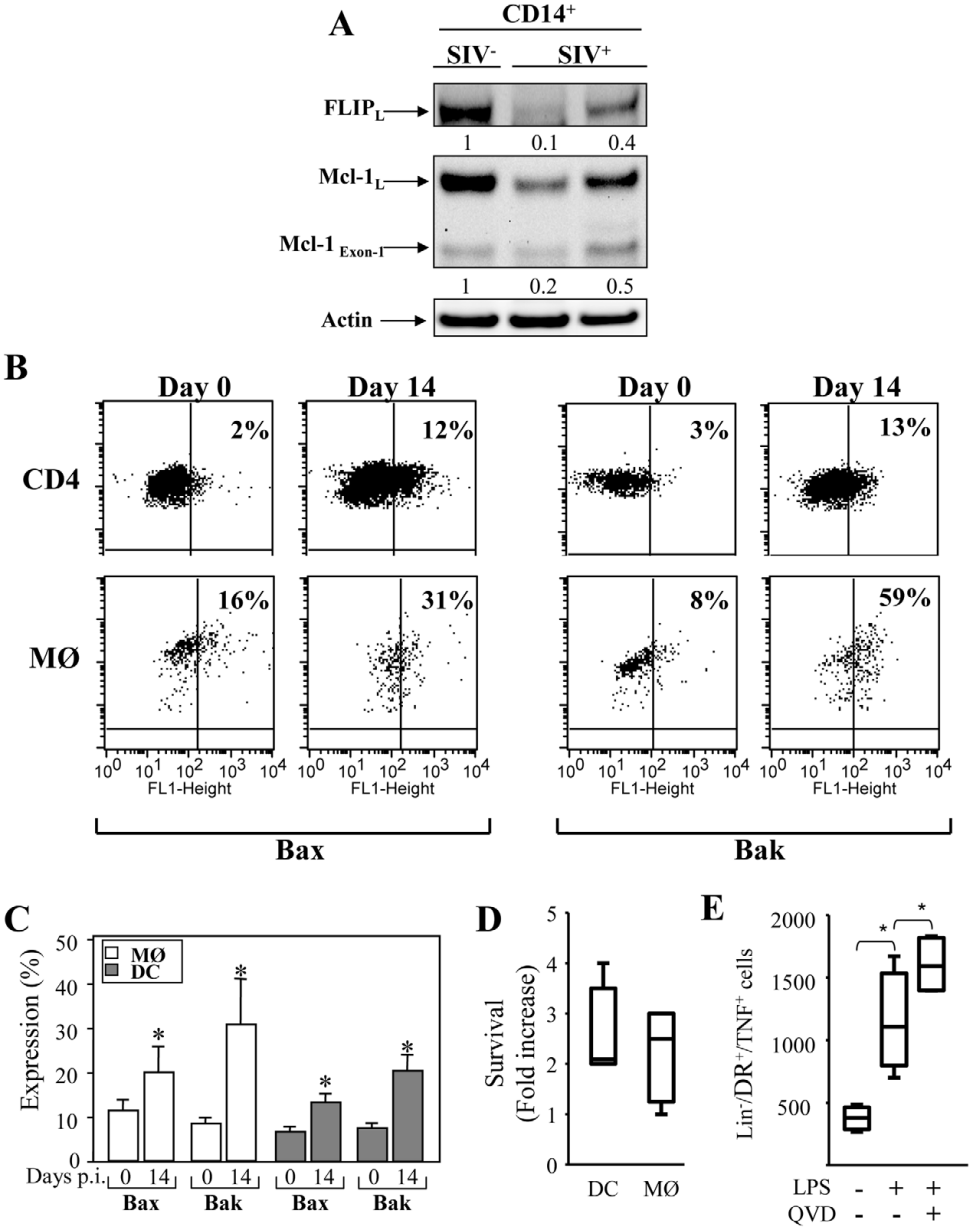
Expression of pro- and anti-apoptotic molecules in monocytes and mDCs during primary SIV infection. (**A**) Expression of FLIP and Mcl-1 in purified CD14^+^ (MØ) from healthy RM (SIV^−^) and SIV-infected RMs (SIV^+^). After isolation, the cells were lysed and the proteins were immunoblotted with specific antibodies against the anti-apoptotic molecules FLIP and Mcl-1. Actin was used as a control for equal protein loading. Values represent the ratio of the FLIP and Mcl-1 bands and normalized with respect to the loading control. (**B**) Flow cytometric analysis of the active form of the pro-apoptotic molecules Bax and Bak in CD4^+^ T cells, and monocytes (MØ) at days 0 and 14. (**C**) Percentage of active form of Bax and Bak among monocyte and DC populations at days 0 and 14. Values are means ± sem (n = 6); Significantly different from day 0 (*, p<0.05). (**D** and **E**) PBMC from SIV-infected RMs were incubated without or with Q-VD-OPH (10 µM) and then stimulated with LPS (10 ng/ml) overnight. (**D**) Fold increase in surviving cells incubated with Q-VD-OPH is shown. (**E**) Numbers of HLA-DR^+^CD3^−^CD20^−^ expressing TNF-α in the absence or presence of Q-VD-OPH after stimulation is shown. Bars show the mean ± SEM of three independent experiments. Statistical significant differences as compared to untreated cells are indicated by an asterisk (p<0.05).

In order to analyze the apoptotic pathways in monocytes, positive selection of CD14^+^ cells was performed from healthy and SIV-infected RMs. Western blots probed with specific antibodies to FLIP revealed that monocytes from SIV-infected RMs displayed lower amounts of FLIP (**Figure 7A**), as compared to healthy RMs. Thus, the absence of FLIP is consistent with the increase sensitivity of these cells to undergo apoptosis after ligation of death receptors. Moreover, we found that monocytes from SIV-infected RMs had lower amounts of Mcl-1 (**Figure 7A**). In one SIV-infected RM, we also detected an increased amount of the proapoptotic form of Mcl-1_Exon-1_. To assess the expression of active form of Bax and Bak proteins in APCs from healthy and SIV^+^RMs, we used specific antibodies that detect conformational changes as previously described [25]. In comparison to CD4^+^ T cells, we found that 20%±6 and 30%±11 of monocytes from SIV-infected RMs at day 14 express the active form of the pro-apoptotic Bax and Bak molecules respectively as compared to monocytes from non-infected RMs (less than 11%±4). Similar data were observed in DCs although to a lesser extent (**Figure 7B and 7C**). Thus, our results indicate that monocyte and DCs are engaged in a process leading to mitochondria damage supporting our observation that these cells are more prone to undergo apoptosis during the acute phase. Furthermore, we used a broad caspase inhibitor and demonstrated that by blocking caspase activation, cell death of APCs was also prevented (**Figure 7D**). We also demonstrated that the addition of caspase inhibitor led to an increase in the number of cells expressing TNF-α after stimulation with LPS + IFN-γ stimulation (**Figure 7E**). Altogether, our data demonstrated a critical role of both the intrinsic and extrinsic apoptotic pathways in controlling APC death during the acute phase of SIV-infection.

## Discussion

We demonstrate that monocytes and DCs are more prone to undergo apoptosis in response to death-receptor ligation after *in vitro* infection with HIV or *ex vivo* from SIV-infected RMs. In addition, our data show that HIV/SIV infection is associated with an increase in the active forms of the pro-apoptotic molecules Bax and Bak and with a decrease in the anti-apoptotic Mcl-1 and FLIP proteins in both cell types. Thus, these results suggest that both the extrinsic and intrinsic pathways are involved in the death of APCs during HIV/SIV infection. Broad inhibition of caspase activation using a synthetic peptide prevented this death and increased the number of TNF-α productive mononuclear cells.

Circulating monocytes are essential not only to replenish the pool of tissue macrophage populations but also may differentiate into inflammatory DCs in the tissues following microbial infection. Because peripheral monocytes and DCs represent crucial populations for the control of pathogens, this enhanced susceptibility to die by apoptosis in the presence of death ligands could have a major impact on the establishment of the adaptative immune response early after infection. Interestingly, other persistent viral infections such as lymphocytic choriomeningitis virus (LCMV) and measles virus (MV), which are associated with a generalized immune suppression in their natural hosts, also induce death of accessory cells early after infection [94], [95]. Our results also demonstrated *in vitro* that incubation of monocyte-derived MØ and DCs with HIV during differentiation not only increased the susceptibility of these cells to undergo apoptosis but also impaired their maturation and their capacity to produce inflammatory cytokines after stimulation. Their down modulation could have an impact on the hosts' ability to mount an effective SIV-specific immune response.

Our results showed abnormal early death of APCs was associated with AIDS. The low level of infection of DCs suggests that apoptosis is not necessarily associated with productive infection. Moreover, during the acute phase, the percentage of monocytes prone to undergo apoptosis (and expressing active form of Bax and Bak) was higher than the frequency of SIV DNA^+^ cells. Our data revealed that also *in vitro* HIV primes both monocytes and DCs to undergo apoptosis in response to death ligands despite the presence of an inhibitor of viral replication, ddI. In a similar manner, the non pathogenic-primate model suggests that despite intense viral replication during the acute phase [70], APCs are not prone to undergo apoptosis. Altogether, these results point to the involvement of indirect mechanisms leading to cell death. This may suggest that triggering of TLRs or other pattern recognition receptors such as a mannose C-type lectin receptor, by HIV could lead to the observed changes in the sensitivity of these cells to undergo apoptosis without active replication [96]. We and others have previously reported the critical role of cytokines determining the sensitivity of monocytes to undergo apoptosis [28], [29], [97], [98]. Among them IL-10 has been shown to be a potent cytokine to induce monocyte death [98] but also increases membrane-bound TNF-α[99], and the expression of CCR5, a co-receptor for SIV [100]. Recently, it has been reported that IL-10 results in the rapid elimination of mature DCs by NK cells but is associated with the accumulation of DCs having an immature phenotype [101]. Blockade of IL-10, in addition to blockade of PD-1 signaling has been suggested as a means to restore anti-viral T cell responses in chronic LCMV infection [102] and to prevent apoptosis [16], [103], [104], [105], [106]. Therefore, whether a therapy based on neutralizing IL-10 antibody would be able to prevent monocyte cell death as well DCs *in vivo* remains an open question.

During inflammation, or in the presence of microbial antigens, monocytes become resistant to death associated with increased expression of anti-apoptotic molecules [28], [29], [31], [32], [107], [108], but resistance to death is counteracted by interferons (IFNs). Indeed, type I IFN production associated with bacterial pathogens such as *Listeria monocytogenes* [109] or in combination with LPS [110] induced apoptosis of APCs. We found that *in vitro* stimulation with LPS + IFN-γ induced the death of HIV-infected APCs as compared to uninfected cells. In HIV-infected patients, a recent study has reported increased levels of monocyte apoptosis after IFN-γ stimulation [66]. Interestingly, bacterial translocation associated with AIDS has been correlated with activation of innate immunity and especially with increased plasma levels of IFNα[111]. Moreover, in pathogenic primate models of SIV infection, increased levels of type I IFN related to the recruitment of pDCs in the lymph nodes (LN) have been reported [91] and associated with disease progression [112]. We found that mononuclear cells were abnormally sensitive to die through apoptosis concomitant with the peak of type I IFN production [91]. Thus, the presence of type I/II IFN may result in increased sensitivity of APCs to undergo apoptosis during the acute phase. After the peaks of viral replication and type I IFN, our data revealed a decrease in the susceptibility of these cells to undergo apoptosis. In chronically HIV-infected persons, a resistance to undergo apoptosis was reported [67]. Therefore, these results together reinforce the idea that death of APCs early after infection contributes to immune deficiency and further progression to AIDS.

Our data clearly demonstrated that *in vitro* and *ex vivo,* APCs were more sensitive to undergo apoptosis in response to specific death-ligands. Monocytes were more prone to die after TRAIL binding than FasL, whereas FasL was more efficacious to induce death in DCs. This increased propensity to undergo apoptosis after death-receptor ligation was not related to an increased expression of the death-receptors at the surface of APC's, but was associated with increased levels of FasL in the sera of SIV-infected RMs and not in non-pathogenic SIV-infected AGMs. Of note, FasL levels in plasma of HIV-positive individuals have been reported to be elevated, and correlated with HIV RNA burden [113], [114]. Elevated levels of TRAIL have also been reported in HIV-infected individuals early after infection [115], [116]. However, our attempts to measure TRAIL in the sera were unsuccessful due to the absence of available reagents for its detection in monkeys. Whereas the incubation of soluble TNF-α had no effect on the death of APCs from RMs, neutralization of TNF-α reduced death. Biologically active forms of death-ligands include both membrane bound and soluble forms, suggesting that TNF-α can be a co-factor for death-receptor sensitization at the cell surface [93]. Taken together, these results suggest that peripheral blood monocytes and DCs from pathogenic SIV-infected macaques are exposed to death-ligands during the acute phase.

Cell death via death receptors can be regulated at different levels, including altered expression of death-ligands or by inhibition of intracellular signaling events. In this context, our results showed that despite death-receptor expression and the presence of death-ligands in the culture, uninfected monocytes and macrophages were resistant to apoptosis indicating that death-ligands and receptors were not sufficient to induce apoptosis. These data are consistent with a model in which infection induced changes in the susceptibility of monocyte and DC populations to undergo apoptosis. The susceptibility of the cells to undergo apoptosis depends on the balance between pro- and anti-apoptotic molecules. We observed a downregulation of FLIP, an inhibitor of the DISC and caspase activation, in HIV-infected MØ- and DCs-derived monocytes. In purified CD14^+^ monocytes from monkeys, we also found a downregulation of FLIP in SIV-infected RMs as compared to healthy RMs. Moreover, the susceptibility of these cells to undergo apoptosis was also associated with increased expression of the active forms of the pro-apoptotic molecules Bax and Bak. Finally, we demonstrated a reduction in the expression of the anti-apoptotic Mcl-1 proteins but its pro-apoptotic form, Mcl-1_Exon-1_, was increased. Thus, our results demonstrated a dysregulation in the balance of pro- and anti-apoptotic molecules, which could contribute to mononuclear phagocyte death. Our results indicated therefore that both the extrinsic and the intrinsic pathways could be closely linked in determining mononuclear cell apoptosis outcome after HIV/SIV infection. In this sense a broad caspase inhibitor prevented cell death. Since monocytes are a heterogeneous population [117], it remains to be determined whether CD14^dim^CD16^+^ monocytes display similar susceptibility to die as CD14^+^ monocytes during HIV/SIV infection.

In conclusion, our findings demonstrate that HIV/SIV infection primes mononuclear cells to undergo apoptosis. Since circulating blood monocytes and DCs extravasate into tissues in response to pathogens, such sensitization to death-receptor mediated apoptosis may be a major factor leading to the defective immune response observed during the acute phase. Taken together, our results highlight the confounding role of apoptosis induction in the physiopathology of HIV/SIV infection associated with the death of mononuclear cells during the acute phase of SIV infection. Thus a strategy aimed at blocking their death could be beneficial in restoring an effective anti-viral response in HIV-infected persons.

## Materials and Methods

### Macaques, virus and samples

Ten RMs (*Macaca mulatta*) seronegative for STLV-1 (Simian T Leukemia Virus type-1), SRV-1 (type D retrovirus), herpes-B viruses, and SIVmac were utilized. RMs were inoculated intravenously with ten 50% animal-infectious doses of the SIVmac251 strain (provided by AM. Aubertin, INSERM U74, Strasbourg, France). Four AGMs of sabaeus species were experimentally infected with 300 TCID50 of SIVagm.sab92018 strain [70].

### Ethics statement

All the animal experiments described in the present study were conducted at the Institut Pasteur according to the European Union guidelines for the handling of laboratory animals (http://ec.europa.eu/environment/chemicals/lab_animals/home_en.htm). The protocol was approved by the committee on the ethics of animal experiments of Ile de France (PARIS 1, #20080007). All surgery was performed under sodium pentobarbital anesthesia, and all efforts were made to minimize suffering.

### Quantitative analysis of frequency of SIV DNA^+^ cells

The frequency of SIV-infected cells was measured by limiting dilution PCR. Cells were isolated from blood and stained with mAbs (CD3, CD4, HLA-DR and CD14 mAbs) (BD Biosciences, San Jose, CA). Cells were purified by cell sorting (FACS Vantage, BD Bioscences) on the basis of their size, granularity and phenotype (CD14^+^HLA-DR^+^CD3^−^CD20^−^; CD14^−^HLA-DR^+^CD3^−^CD20^−^ versus CD3^+^CD4^+^); purity exceeded 98%. Purified cells were counted and serially diluted in a constant number of carrier CEMX174 cells as previously described [22]. The proportion of SIV DNA^+^ cells in purified cells was determined using Poisson law. The limiting-dilution PCR method detected one SIV DNA^+^ cell in 10,000 uninfected cells (CEMX174) validated with SIV-1C cells (provided by F. Villinger), that contain a single provirus of SIVmac251 per cell.

### Cell culture

Fresh PBMC were isolated by density gradient centrifugation from blood of healthy donors. The blood samples were obtained from the Institut National de la Transfusion Sanguine. Monocytes were obtained by plastic adherence after extensive washing with media to remove non-adherent cells as described [98]. The adherent monocytes were carefully removed from the culture by incubating the plate 30 min at 4°C and use cold PBS and pipetting (not scraping). The purity of the monocytes exceed 90–95% as determined by flow cytometry after cell staining with mAbs CD14, CD3, CD20 and HLA-DR. The cells were then incubated in RPMI-1640 supplemented with 10% FCS, 1% glutamine, 1% pyruvate, and 1% antibiotics. Macrophages were derived from monocytes in the presence of GM-CSF (10 ng/ml) and IL-6 (5 ng/ml) (R&D system) while dendritic cells were derived in the presence of GM-CSF and IL-4 (10 ng/ml). At day one, the cells were incubated in the presence of the R5 HIV-1BaL strain (100 pg/ml of p24). At day 5, the cells were stimulated overnight with LPS (10 ng/ml) and IFN-γ (10^3^ U/ml). Cells were also incubated in the presence or absence of TNF-α, TRAIL and FasL (200 ng/ml). Fresh PBMC from Non Human primates were isolated by density gradient centrifugation from blood and use to perform the different assays [21], [25], [88].

### Quantitation of viral infection

Infections of monocyte-derived MØ and DCs, respectively, were measured by flow cytometry based on the detection of intracellular p24 antigen (RD1-labeled mAb anti-p24 antigen, KC-57, Beckman coulter) after fixation and permeabilization of the cells (Intraprep permeabilization reagent, Coulter Coultronics). Productive HIV infection was also visualized by western blotting that allows detection of the presence of viral antigens in cell extracts. The immunoblots were incubated with sera obtained from a pool of HIV^+^ infected patients (kindly provided by F Mamano, Institut Pasteur). After treatment with horseradish peroxidase-linked goat anti-human secondary antibodies (Amersham Biosciences), immunoreactive proteins were detected using enhanced chemiluminescence (ECL^+^ from GE Healthcare) using a CCD camera (GBOX, SYNGENE).

The frequencies of SIV-infected CD4^+^ T cells, CD14^+^ and DCs were measured by limiting-dilution PCR [22] of purified cells by cell sorting (FACS Vantage; Becton Dickinson Biosciences, Le Pont de Claix, France) using the positive selection of cells stained with specific antibodies. Purified cells were counted and diluted in series in a constant number of carrier CEM X 174 cells. Cells were directly lysed with TPK buffer (10 mM Tris-HCl pH 8.3, 50 mM potassium chloride, 2.5 mM magnesium chloride, 0.5% Nonidet P-40, 0.5% Tween 20, 100 µg/ml of proteinase K). After 1 h at 56°C, proteinase K was inactivated at 95°C for 10 min. Twenty replicates of limiting dilutions were submitted to a nested PCR. SIV proviral DNA was amplified by nested PCR with SIV251-specific primers surrounding the nef region. After 35 cycles (95°C for 30 s, 60°C for 30 s, 72°C for 1 min.) with the first set of primers, Preco (59-CAG AGG CTC TCT GCG ACC CTA C) and K3 (59-GAC TGA ATA CAG AGC GAA ATG C), amplified a fragment of 961 base pairs, 10 µl of product was re-amplified (30 cycles 95°C for 30 s, 55°C for 30 s, 72°C for 1 min) with primers K1 (59-TGG AAG ATG GAT CCT CGC AAT CC) and A2 (59-GGA CTA ATT TCC ATA GCC AGC CA). Nested PCR products were electrophoresed through a 1.8% agarose gel. The proportion of infected cells was determined using Poisson law. The limiting-dilution PCR method was able to detect one infected cell in 10 000 uninfected cells (CEM X 174) demonstrated with SIV-1C cells (provided by F. Villinger), which contain a single provirus of SIVmac251 per cell.

### Pro-inflammatory cytokine assays

Supernatants were collected after overnight stimulation and 6 days of culture. IL-1ß, IL-6, IL-8, and TNF-α were detected simultaneously by using the human inflammatory cytokine cytometric bead array (CBA) kit (BD Bioscience) [90]. The CBA working range was 20–5000 pg/ml for each cytokine. Cytokine levels were quantified by flow cytometry according to the manufacturer's directions. For intracellular TNF-α staining, the cells were incubated in the absence or presence of a broad caspase inhibitor Q-VD(OMe)-OPH (10 µM, MBL biomedical), and stimulated with LPS and IFN-γ. After 8 h, the cells were first stained with HLA-DR, CD3, and CD20 mAbs, washed and then permeabilized, before staining with PE-TNF-α mAbs (BD Biosciences). The number of HLA-DR^+^CD3^−^CD20^−^ cells expressing TNF-α was measured by flow cytometry.

### Quantitation of FasL in the sera of SIV-infected monkeys

FasL in the serum was measured using a solid-phase immunoassay (MBL). The assay uses anti-FasL mAbs (clones, 4H9 and 4A5). The peroxidase substrate was used to quantify FasL and the optical density measured at 450 nm. The concentration was determined using a standard curve based on recombinant FasL. Three distinct ELISA specific for human TRAIL purchased from R&D system, Diaclone and Kamiya Biomedical Company, however, were unable to detect monkey Trail in the sera/plasma.

### Immunophenotyping of APCs

Monocyte-derived MØ and DCs, respectively, cultured in the absence or presence of the R5 HIV-1_Bal_ strain, were then incubated in the absence (medium) or presence of LPS (10 ng/ml) plus IFN-γ (10^3^ U/ml). Cells were then stained with FITC-CD14, APC-CD11c, PerCP-HLA-DR, and PE-conjugated antibodies to either CD83 (HB15e) or CD86 molecules (FUN-1) (BD Biosciences). Five hundred thousand events corresponding to mononuclear cells were acquired using a FACScalibur instrument (BD Biosciences).

### Quantitative assessment of dead cells after HIV/SIV infection

Fresh PBMC from SIV-infected RMs at different days post-infection were isolated by density gradient centrifugation; apoptosis of monocytes and dendritic cells was determined at 24 h of culture by cell surface staining and with FITC-labeled annexin-V which is an early marker of dying cells detecting both caspase-dependent and -independent cell death programs [118]. The level of apoptosis was determined by flow cytometry as previously described [98]. We also used decoy receptors of Fas (Fas-Fc), TRAIL (TRAIL-R1-Fc and TRAIL-R2-Fc) and TNF-α (TNF-R1-Fc) at a dose of 10 µg/ml (Alexis corporation) as previously described [21].

### Expression of pro- and anti-apoptotic molecules

Cells from SIV-infected RMs and healthy RMs were first labeled for cell surface markers (APC-HLA-DR, PE-CD14, Lineage PerCP-CD3/CD20 versus APC-CD11c, PE-HLA-DR and Lineage PerCP-CD3/CD20) and then fixed and permeabilized. Cells were then incubated with anti-Bax (BD Biosciences) or anti-Bak Abs (Calbiochem) as previously described for primates [25]. After washing, FITC-labeled goat anti-rabbit IgG Ab (Molecular probes) was added for 30 min at 4°C in the presence of mouse immunoglobulins. Cells were then washed and analyzed by flow cytometry.

Pellets of 3×10^6^ monocyte-derived MØ and DCs, respectively, were lysed in Nonidet P-40 buffer (1% NP-40, 50 mM Tris-HCl (pH 7.4), 150 mM NaCl) containing protease and phosphatase inhibitors. Total lysates were resolved by SDS-PAGE (10–20% Tricine gels, Novex) and transferred to nitrocellulose membranes (Amersham Biosciences). After blocking nonspecific sites for 1 hour at room temperature with 5% nonfat milk and 0.2% Tween 20 in phosphate-buffered saline (pH 7.4), the membrane was incubated with rabbit anti-Bak (Calbiochem, clone 2–14), rabbit polyclonal anti-Bax (Santa-Cruz, N-20), rabbit anti-Mcl-1 (S19, Santa-Cruz), or rat anti-FLIP (DAVE-2, Alexis Corporation). To confirm equal protein loading and transfer, membranes were reprobed with anti-actin monoclonal antibodies (Sigma). After treatment with horseradish peroxidase-linked goat anti-mouse or anti-rabbit secondary antibodies (Amersham Biosciences), immunoreactive proteins were detected using enhanced chemiluminescence (ECL^+^ from GE Healthcare) using a CCD camera (GBOX, SYNGENE).

### Statistical analyses

Data are reported as means ± SEM, and groups were compared using Mann-Whitney test (Prism software, GraphPad, San Diego CA). A p value <0.05 was considered significant.
